# Supplementary material for: Toll-like Receptor 2 Mediates VEGF Overexpression and Mesothelial Hyperpermeability in Tuberculous Pleural Effusion
Source: Int J Mol Sci. 2023 Feb 2;24(3):2846. doi: 10.3390/ijms24032846 (PMC9918151; doi:10.3390/ijms24032846)
Supplement: Supplementary file 1 [file ijms-24-02846-s001.zip › Figure S1.pdf]

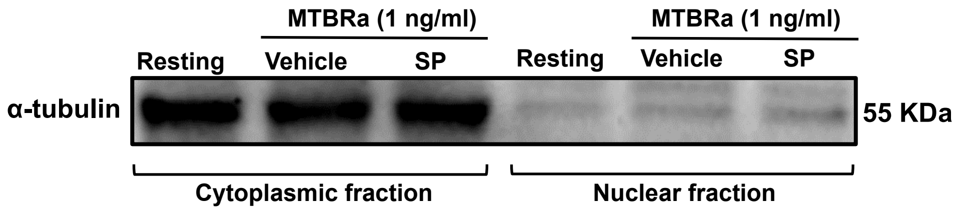

Figure S1. Verification of no cytoplasmic contamination in the isolated nuclear fraction of MeT-5A human pleural mesothelial cells treated with vehicle or SP 10 μM followed by MTBRa treatment for 15 min. The cytoplasmic contamination in the isolated nuclear faction was determined by western blotting, using α-tubulin as the cytoplasmic probe. SP, SP600125.
