# Supplementary material for: Toll-like Receptor 2 Mediates VEGF Overexpression and Mesothelial Hyperpermeability in Tuberculous Pleural Effusion
Source: Int J Mol Sci. 2023 Feb 2;24(3):2846. doi: 10.3390/ijms24032846 (PMC9918151; doi:10.3390/ijms24032846)
Supplement: Supplementary file 1 [file ijms-24-02846-s001.zip › Figure S2.pdf]

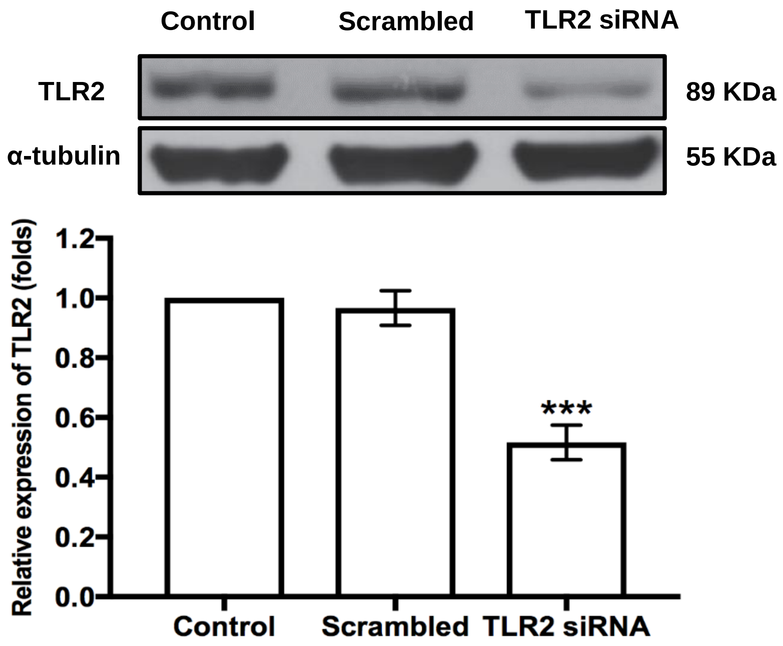

Figure S2. MeT-5A human pleural mesothelial cells were transfected with scrambled siRNA or TLR2 siRNA (25 nM) for 48 hours, then TLR2 and α-tubulin proteins were determined by western blotting. Data are representative of three separate experiments. *** $p < 0.001$ compared with scrambled siRNA group.
