# Supplementary material for: Toll-like Receptor 2 Mediates VEGF Overexpression and Mesothelial Hyperpermeability in Tuberculous Pleural Effusion
Source: Int J Mol Sci. 2023 Feb 2;24(3):2846. doi: 10.3390/ijms24032846 (PMC9918151; doi:10.3390/ijms24032846)
Supplement: Supplementary file 1 [file ijms-24-02846-s001.zip › Table S1.pdf]

Table S1. Raw denstiometric data

A

	R	0.1	1	10
VEGF	1	0.983	1.647	1.32
Tubulin	1	0.943	1.086	1.064
	1.00	1.04	1.52	1.24

	R	0.1	1	10
VEGF	1	1.273	1.724	1.18
Tubulin	1	0.953	1.025	0.983
	1.00	1.34	1.68	1.20

	R	0.1	1	10
VEGF	1.00	1.43	1.53	1.11
Tubulin	1.00	0.91	0.93	1.02
	1.00	1.56	1.64	1.09

B

	R	V	Par	LY	PD	SB	SP
VEGF	1	1.404	1.284	1.447	1.181	1.043	0.699
Tubulin	1	1.006	0.841	0.926	0.836	0.975	1.105
	1.00	1.40	1.53	1.56	1.41	1.07	0.63

	R	V	Par	LY	PD	SB	SP
VEGF	1	1.451	1.482	1.684	1.496	1.623	1.304
Tubulin	1	0.94	1.012	1.072	1.079	1.22	1.4
	1.00	1.54	1.46	1.57	1.39	1.33	0.93

	R	V	Par	LY	PD	SB	SP
VEGF	1	1.364	1.616	1.293	1.739	1.368	0.825
Tubulin	1	0.921	1.068	1.022	0.996	1.038	0.989
	1.00	1.48	1.51	1.27	1.75	1.32	0.83

C

	R	1	5	15	30
P-JNK	1	0.95	1.98	1.49	0.74
T-JNK	1	0.95	0.98	0.95	1.05
	1.00	1.00	2.02	1.57	0.70

	R	1	5	15	30
P-JNK	1	0.95	1.84	1.25	0.66
T-JNK	1	0.78	0.9	0.78	0.9
	1.00	1.22	2.04	1.60	0.73

	R	1	5	15	30
P-JNK	1	1.51	1.88	2	0.82
T-JNK	1	1.06	1.09	1.08	1.27
	1.00	1.42	1.72	1.85	0.65

D

	R	Vehicle	SP 10
VEGF	1	1.936	1.907
GAPDH	1	0.973	1.015
	1.00	1.99	1.88

	R	Vehicle	SP 10
VEGF	1	2.331	1.937
GAPDH	1	0.948	1.025
	1.00	2.46	1.89

	R	Vehicle	SP 10
VEGF	1	2.238	2.129
GAPDH	1	0.948	1.025
	1.00	2.36	2.08
